# Genetic interactions regulate hypoxia tolerance conferred by activating Notch in excitatory amino acid transporter 1-positive glial cells in *Drosophila melanogaster*

**DOI:** 10.1093/g3journal/jkab038

**Published:** 2021-01-28

**Authors:** Dan Zhou, Tsering Stobdan, DeeAnn Visk, Jin Xue, Gabriel G Haddad

**Affiliations:** 1 Division of Respiratory Medicine, Department of Pediatrics, University of California San Diego, La Jolla, CA 92093, USA; 2 Department of Neurosciences, University of California at San Diego, La Jolla, CA 92093, USA; 3 Rady Children’s Hospital, San Diego, CA 92123, USA

**Keywords:** Notch signaling, genetic interactions, eaat1-posive glia, Hypoxia, *Drosophila melanogaster*

## Abstract

Hypoxia is a critical pathological element in many human diseases, including ischemic stroke, myocardial infarction, and solid tumors. Of particular significance and interest of ours are the cellular and molecular mechanisms that underlie susceptibility or tolerance to low O_2_. Previous studies have demonstrated that Notch signaling pathway regulates hypoxia tolerance in both *Drosophila melanogaster* and humans. However, the mechanisms mediating Notch-conferred hypoxia tolerance are largely unknown. In this study, we delineate the evolutionarily conserved mechanisms underlying this hypoxia tolerant phenotype. We determined the role of a group of conserved genes that were obtained from a comparative genomic analysis of hypoxia-tolerant *D.melanogaster* populations and human highlanders living at the high-altitude regions of the world (Tibetans, Ethiopians, and Andeans). We developed a novel dual-UAS/Gal4 system that allows us to activate Notch signaling in the Eaat1-positive glial cells, which remarkably enhances hypoxia tolerance in *D.melanogaster*, and, simultaneously, knock down a candidate gene in the same set of glial cells. Using this system, we discovered that the interactions between Notch signaling and *bnl* (fibroblast growth factor), *croc* (forkhead transcription factor C), or *Mkk4* (mitogen-activated protein kinase kinase 4) are important for hypoxia tolerance, at least in part, through regulating neuronal development and survival under hypoxic conditions. Becausethese genetic mechanisms are evolutionarily conserved, this group of genes may serve as novel targets for developing therapeutic strategies and have a strong potential to be translated to humans to treat/prevent hypoxia-related diseases.

## Introduction

Hypoxia (O_2_ deprivation) is a common pathological factor in many human diseases, including apnea of prematurity, hypoxemia in ICU settings resulting from neurologic, hematologic, respiratory and cardiac diseases, stroke, and cancer (Sands and Owens 2015; Adler *et al.* 2017; Schmidt *et al.* 2017, 2019; Group 2018; Thille *et al.* 2018; Di Fiore *et al.* 2019). To date, strategies to treat or prevent hypoxia-induced injury are very limited. Thus, understanding the mechanisms regulating tolerance or susceptibility to hypoxia is crucial for developing effective therapeutic strategies.

A number of studies have demonstrated that Notch signaling plays an important role in regulating the cellular and molecular mechanisms underlying the responses to hypoxic stress (for selected reviews, see Andersson *et al.* 2011; Alberi *et al.* 2013; Marignol *et al.* 2013; Zhou and Haddad 2013; Borggrefe *et al.* 2016; Fearon *et al.* 2016; Arumugam *et al.* 2018). For example, (1) it has been shown that Notch intracellular domain coordinates with HIF signaling to regulate cellular response to hypoxia (Gustafsson *et al.* 2005; Pear and Simon 2005; Sainson and Harris 2006); (2) we have previously discovered that Notch signaling is activated and plays a critical role in regulating hypoxia tolerance in *Drosophila melanogaster* (*i.e., D.melanogaster* carrying *Notch* loss-of-function alleles are super-sensitive to low O_2_, and, in contrast, the fruit flies carrying gain-of-function alleles are remarkably resistant) (Zhou *et al.* 2008, 2011) (3) neuronal- or glial-specific activation of Notch rescues naïve flies from lethal degree of O_2_ deprivation (Zhou *et al.* 2011); and (4) Notch signaling is an evolutionarily conserved mechanism regulating adaptation to low O_2_ environments not only in *Drosophila* but also in humans (Jha *et al.* 2016). However, the molecular mechanisms underlying the role of Notch signaling in regulating hypoxia responses are still not well understood. BecauseNotch is highly pleiotropic, it is clear that specific downstream responses to Notch activation depend on cellular context and its synergic integration with other signaling pathways (Bray and Bernard 2010; Ho *et al.* 2018). Furthermore, due to the broad physiological role of Notch signaling in cell differentiation and metabolism, therapeutic strategies directly targeting *Notch* may introduce severe side-effects (Rizzo *et al.* 2014; Takebe *et al.* 2014; Mollen *et al.* 2018). Therefore, identifying genetic modifiers or downstream effectors that mediate the role of Notch in hypoxia are very important for developing effective therapeutic strategies.

It has long been recognized that hypoxia does not affect all organs/tissues of the body equally. Of all the organs, brain is one of the organs that are very sensitive to oxygen deprivation (Leach and Treacher 1998; Purins *et al.* 2012). We previously discovered that hypoxia-induced lethality can be rescued by activating Notch signaling only in the excitatory amino acid transporter 1 (Eaat1)-positive glial cells in *D.melanogaster*, which is likely through cell injury prevention in the central nervous system. It is remarkable that the activation of Notch in this group of glial cells alone is sufficient to enhance the organismal survival under prolonged hypoxic conditions (Zhou *et al.* 2011). Presumably, this group of Eaat1-positive glial cells regulates hypoxia tolerance through suppression of glutamate neurotoxicity, neuronal proliferation as well as the growth and organization of axons (Gibson 1989; Ikonomidou *et al.* 1989; Stacey *et al.* 2010). Furthermore, since we have identified a group of evolutionarily conserved genes and biological processes (including Notch signaling) between *D.melanogaster* and human highlanders (Jha *et al.* 2016) that are important for survival in hypoxic environment, some of these conserved genes may interact with Notch signaling to regulate Notch activation-conferred hypoxia tolerance. In this study, we studied the genetic interactions between Notch signaling and a group of these conserved genes [*i.e.,branchless* (*bnl*), *crocodile* (*croc*), *Epidermal growth factor receptor* (*Egfr*), *grain* (*grn*), *hairy* (*h*), *invected* (*inv*), and *MAP kinase kinase 4* (*Mkk4*)]and identified modifiers that regulate Notch activation-conferred hypoxia tolerance in *D.melanogaster*.

## Materials and methods

### Drosophila stocks and culture conditions

The following available TRiP UAS-RNAi lines, UAS-reaper lines and Gal4 driver stocks were obtained from the Bloomington *Drosophila* Stock Center: [y^1^ sc^*^ v^1^ sev^21^; P{*TRiP.HMS01046*}*attP2*] (bnl-RNAi, FBst0034572), [y^1^ sc^*^ v^1^ sev^21^; P{*TRiP.HMS01122*}*attP2*] (croc-RNAi, FBst0034647), [*y^1^ v^1^; P*{*TRiP.JF02283*}*attP2*] (Egfr RNAi, FBst0036770), [y^1^ sc^*^ v^1^ sev^21^; P{*TRiP.HMS01085*}*attP2*] (grn-RNAi, FBst0033746), [y^1^ sc^*^ v^1^ sev^21^; P{*TRiP.HMS01052*}*attP2*] (grn-RNAi, FBst0034578), [y^1^ sc^*^ v^1^ sev^21^; P{*TRiP.HMS01313*}*attP2*] (h-RNAi, FBst0034326), [y^1^ sc^*^ v^1^ sev^21^; P{*TRiP.HMS02209*}*attP2*] (inv-RNAi, FBst0041675), [y^1^ sc^*^ v^1^ sev^21^; P{*TRiP.HMS02524*}*attP40*] (Mkk4-RNAi, FBst0042832), [*w^1118^; P*{*UAS-rpr.C*}*27*] (FBst0005823), [*w^1118^; P*{*UAS-rpr.C*}*14*] (FBst0005824), [w^*^; Kr[*If-1*]/*CyO; P*{*w*[*+mW.hs*]*=GAL4-da.G32*}*UH1*] (FBst0055850, was used to derive da-Gal4) and [w^*^; P{*w*[*+mC*]*=Eaat1-GAL4.R*}*2*] (Eaat1-Gal4, FBst0008849). The UAS-NICD and 4XSu(H)-lacZ stocks were kindly provided by Dr. J. Posakony. BecauseUAS-NICD transgenic stock was generated on the background of *w^1118^*(Go *et al.* 1998), the *w^1118^* stock (FBst0003605) was used as one of the negative controls in the dual-UAS/Gal4 experiments.

Flies were maintained in vials with Cornmeal-Molasses-Yeast medium. Flies for the hypoxia tolerance assay were prepared as follows. 20 Females of UAS-RNAi were crossed with 20 males of EN line, Eaat1-Gal4 or da-Gal4 and incubated at room air/temperature condition. After 24 hours, parents were removed. One group of the vials (*n *= 3 to 6 vials) containing the embryos were transferred to a computerized atmosphere chamber supplied with 5% oxygen for hypoxia treatment, and the other group of vials (*n *= 3 to 6 vials) were cultured in room air condition and used as controls.

### Generation of dual-UAS/Gal-4 system

We have generated a unique fly line that has Notch upregulated specifically in the glial cells that produce the glutamate transporter EAAT1. The details on the strategy to generate this line are provided in Supplementary Figure S1. Briefly the UAS-NICD inserted on 3rd chromosome and Eaat1-Gal4 ([w*; P{*w*[*+mC*]=*Eaat1-GAL4.R*}*2*] (FBst0008849); stock# 8849, Bloomington, USA) were simultaneously crossed with a double balancer [w*; Cyo; TM3, Sb’](stock#2475, Bloomington, USA). The F1 flies with both *Cyo* and *Sb* phenotype from both crosses (*i.e.*, [*w; +/Cyo; UAS-NICD/TM3, Sb’*]from the first cross and [*w;P*{*Eaat1-GAL4.R*}2/*Cyo; +/TM3, Sb’*]from the second cross) were selected and self-crossed to remove flies with *Sb’* phenotype from the [*w; +/Cyo; UAS-NICD*/*TM3, Sb’*]line and *Cyo* phenotype from the [*P*{*Eaat1-GAL4.R*}2/*Cyo;+*/*TM3, Sb’*]lines. Subsequently, these flies were intercrossed and again select for flies with *Cyo* and *Sb’* phenotype. Finally, they were self-crossed to obtain homozygote [*w; P*{*Eaat1-GAL4.R*}2*; UAS-NICD*] (*i.e.*, the EN line).

### Hypoxia treatment and survival test

Three- to five-day-old Eaat1-Gal4and EN males (*n *=  10) were crossed to the UAS-RNAi virgin females (*n *=  10) targeting specific genes. Sufficient time was given (∼3 days) for the flies to mate/cross. Simultaneously the *w^1118^*and the Gal4 line were ‘self-crossed’ and used as negative controls, the [*EN*] x [*w^1118^*] cross was used as a positive control. Each set of crosses were in 3 to 6 replicate vials. The vials were kept under ambient conditions for 48 hours so that the flies can lay sufficient number (50 to 100) of eggs. After 48 hours, the adults were transferred to a new vial. For the hypoxia tolerance test the original vials were then transferred to a computer-controlled hypoxia chamber, constantly maintained at 5% oxygen. The chambers were in a room with 12/12 hours light/dark cycle, and temperature ∼22^°^C. The adults from the new vials (*i.e.*, from the second batch of vials) were discarded after 48 hours, and the vials with the eggs were kept at ambient oxygen conditions (∼21% O_2_) also with 12/12 hours light/dark cycle (temperature ∼22^°^C) as room air controls. After 21 days, the ratio of the empty pupae (eclosed) to the total number of pupae formed (eclosed + uneclosed) in each vial was calculated to determine the eclosion rate. Due to the fact that most of the collected embryos will be at an early larval stage, this hypoxia test was mainly to determine the survival rate from larval stage to adulthood, as representated by eclosion rate.

### Immunostaining and microscopy

The third instar larval brain samples for immunostaining were prepared according to previous descriptions (Wu and Luo 2006; Zhou *et al.* 2011). Briefly, brains of wandering third instar larvae were dissected in PBS and fixed in 4% paraformaldehyde in PBS. Cell membranes were permeabilized with 0.3% Triton X-100 in PBS, blocked with 7% goat serum, and put in primary antibody overnight at 4°C (or for 1 hour at room temperature). This procedure was followed by washes in 0.3% Triton X-100 in PBS, incubation with secondary antibody for 90 minutes. Following staining, the brain samples were washed multiple times with PBS, mounting and microscopy. The mouse anti-NICD (undiluted in-house supernatant) and rat anti-elav (1:50) were obtained from the Developmental Studies Hybridoma Bank (DSHB); rabbit anti-repo (1:500) was a kind gift of Dr. G. Technau at the Institute of Genetics, University of Mainz, Mainz, Germany. Secondary antibodies used were goat anti-mouse; goat anti-rabbit; and goat anti-rat conjugated to Alexa 488, 546, or 647 (1:250, Invitrogen). The Prolong Gold anti-fade reagent with DAPI (Invitrogen) was used as mounting media. Confocal microscopy was performed in the University of California at San Diego Neuroscience Microscopy Shared Facility. Imaging was done on a confocal microscope (Olympus FV1000) and the images were processed with Image J.

### Statistical analysis

Data were analyzed and graphed using GraphPad Prism 6 software. The differences in eclosion rate at 5% oxygen between the [*EN*]×[*UAS-RNAi*]and [*Eaat1-Gal4*]×[*UAS-RNAi*]and all the controls were assessed using ordinary one-way ANOVA with Turkey’s multiple comparison tests.

## Results and discussion

The Eaat1-positive cells are a group of glia regulating glutamate metabolism and transport as well as neuronal activity in *Drosophila* brain (Rival *et al.* 2004; Peco *et al.* 2016). We have previously discovered that hypoxia activates Notch signaling (Zhou *et al.* 2008), and this activation particularly in the Eaat1-positive glial cells can significantly enhance survival of *Drosophila* in severely hypoxic environments (Zhou *et al.* 2011). We aimed in this study to dissect the potential mediators of Notch-conferred hypoxia tolerance.

### Eaat1-positive glial cells are critical for *Drosophila* development and survival

Glutamate is both the principal excitatory neurotransmitter and a potent neurotoxin (at high concentrations) in the mammalian CNS (Krnjevic 1970; Olney and Ho 1970; Fonnum 1984; Choi 1988; Nicholls 1993). Indeed, extracellular glutamate levels are tightly regulated for precise control of neurotransmission at glutamatergic synapses, and to prevent neuronal cell death from excitotoxicity (Danbolt 2001). In *Drosophila*, this process is regulated by a group of cells that express Eaat1, the only *Drosophila* high-affinity glutamate transporter (Besson *et al.* 1999). Although a previous study in adult flies has shown that RNAi-mediated knocking down of *Eaat1* in glial cells increased sensitivity to oxidative stress, enhanced degeneration of the brain neuropil and decreased lifespan (Rival *et al.* 2004), the importance of this group of glial cells in neuronal development and function still remains largely unknown.

**Figure 1:**
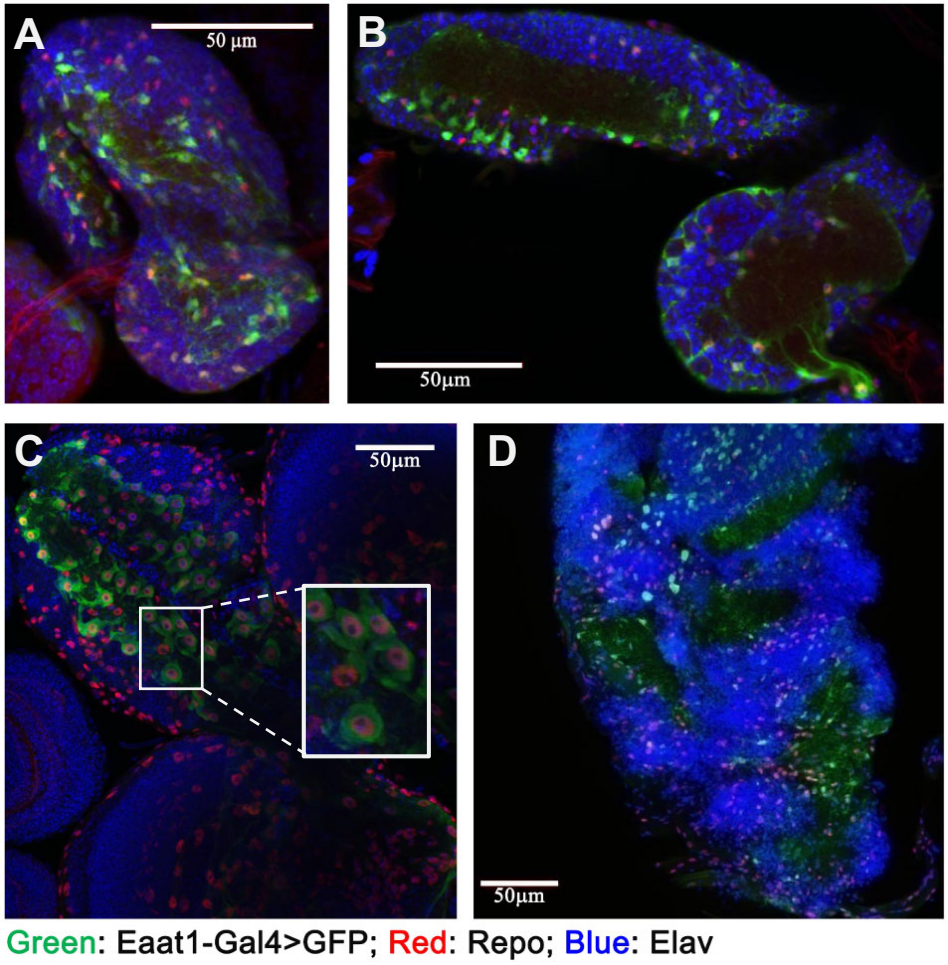
Distribution of Eaat1-positive glial cells during development. The Eaat1-positive glial cells were labeled by GFP in the progeny of [*Eaat1-GAL4*]×[*UAS-GFP*]crosses. The expression of glial marker (repo) and neuronal marker (elav) were labeled by immunostaining. (A–D): the distribution of Eaat1-positive glial cells at first (A), second (B), and third (C) instar larval as well as adult (D) brain. Colocalization of GFP and Repo within the same cell was highlighted in (C) (insert, image represents a Z-projection of 5–12 slices). GFP (green), Repo (red), and Elav (blue). Scale bar = 50 µm.

**Figure 2:**
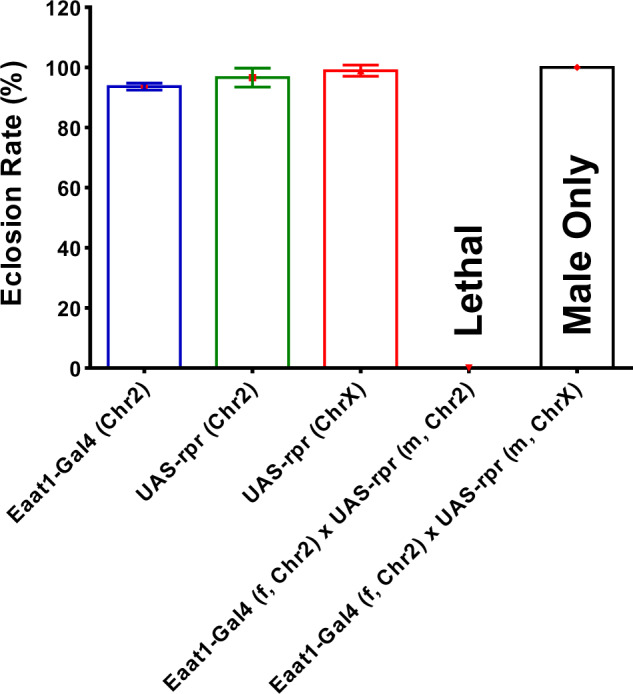
Eaat1-positive glial cells are important for the development and survival of *D.melanogaster*. *Eaat1-Gal4* virgin female was crossed with male *UAS-rpr* with transgene inserted on 2nd or X chromosome (Chr2 or ChrX) to deplete the Eaat1-positive glial cells by rpr-induced cell death. The crosses of *Eaat1-Gal4* with *UAS-rpr* (m, Chr2) were lethal in both males and females, none of the embryos reached adult stage. In the cross between female *Eaat1-Gal4* and male *UAS-rpr* (m, ChrX), only male flies were obtained, demonstrating that the Eaat1-positive glial cells are essential for the development and survival of *D.melanogaster*.

First, we determined the developmental expression pattern of *Eaat1* by crossing Eaat1-Gal4 with UAS-GFP. As shown in Figure 1, the Eaat1-Gal4 expressing cells were detected throughout all developmental stages from first instar larvae to adulthood. The Eaat1-Gal4 positive cells are a subgroup of glial cell that co-express the glial marker Repo (Figure 1C). Then, to further determine the role of Eaat1-positive glial cells in development and survival of *D.melanogaster*, we ablated these cells by specific expression of an apoptotic gene *reaper* (*rpr*) in the Eaat1-positive glial cells through crossing the virgin female Eaat1-GAL4 with male UAS-rpr carrying the UAS-rpr transgene on the 2nd or the X chromosome. We found that depletion of this group of cells in both males and females (in the progeny of Eaat1-Gal4 female crossed with male UAS-rpr carrying transgene on the 2nd chromosome) terminated *D.melanogaster* development at embryonic stage under room air condition, as no pupae and adult flies were obtained. To further confirm this phenotype, we crossed the Eaat1-Gal4 female with male UAS-rpr carrying the transgene on the X chromosome. With this cross, the *rpr* transgene was only expressed in the female progeny. As expected, only male progeny survived (Figure 2), demonstrating that these glial cells are essential in organismal development and survival.

Neurons and glia are two major cell types in the nervous system of both vertebrate and invertebrate animals. Glial cells are critical for the development and function of neurons by providing neurons with survival and axonal guidance cues, electric shield, and acting as macrophages to remove injured/dead cells (Yildirim *et al.* 2019; Bittern *et al.* 2020; Hilu-Dadia and Kurant 2020). Previous studies have shown that Notch signaling plays an important and evolutionarily conserved role in regulating glial differentiation and proliferation during development or following neuronal injuries (Jacobs 2000; Hidalgo and Logan 2017; Kato *et al.* 2018; Bahrampour and Thor 2020). Our previous study has shown that increasing Notch activity specifically in this set of glial cells dramatically enhanced hypoxia survival, implying that these glial cells also play an important role in regulating organismal development and survival under stress conditions (Zhou *et al.* 2011), which, at least in part, through rebalancing hypoxia-induced dysregulation and neurotoxicity of glutamate, maintaining the function of neuropil and development of neuronal-glial system (Choi and Rothman 1990;Stacey *et al.* 2010; MacNamee *et al.* 2016). In addition, discovery of the property of Notch signaling in regulating cellular responses to hypoxic challenge further broadened the pleiotropic nature of this signaling pathway (Zhou *et al.* 2011; Arumugam *et al.* 2018).

### Creation and characterization of a dual-UAS/Gal4 system

**Figure 3:**
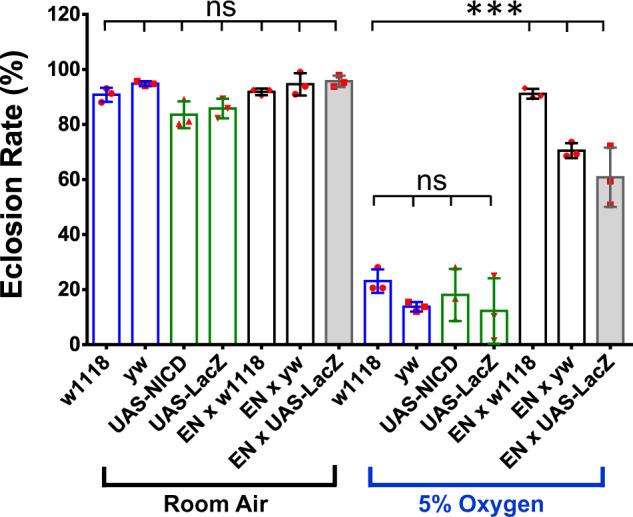
Characterization of the dual-UAS/Eaat1-Gal4 system. The EN line was crossed with UAS-LacZ or the background *D.melanogaster* strains (*w^1118^* or *yw*) for the transgenic lines to test the efficiency of this system. No alterations on survival were observed in the crosses and controls under room air condition. A significant enhancement of hypoxia survival was detected in the [*EN*] × [*w1118*], [*EN*] × [*yw*], and [*EN*] × [*UAS-LacZ*] as compared to the controls (*w1118*, *yw*, and *UAS-LacZ*) under 5% O_2_, demonstrating that double the dosage of UAS-transgenes Eaat1-Gal4 > UAS-NICD/UAS-LacZ (the progeny of [*EN*] × [*UAS-LacZ*]) did not significantly affect the hypoxia tolerant phenotype showing in Eaat1-Gal4 > UAS-NICD (the progeny of [*EN*] × [*w1118*] or [*EN*] × [*yw*]). Bars represent mean ± SD (*n *= 3 vials) for each group/treatment. Ordinary one-way ANOVA [*F*_(13, 28)_= 108.3, *P* < 0.0001]with Turkey’s multiple comparison tests (*** *P* < 0.001; ns: not significant).

In order to determine genetic interactions that mediate the function of Notch signaling under hypoxic conditions, we created a dual-UAS/Gal4 system (Supplementary Figure S1). This system contains an Eaat1-Gal4 transgene that simultaneously drives the expression of a UAS-NICD (the functional domain of Notch receptor) transgene to activate Notch signaling and a UAS-RNAi transgene to target and knockdown the expression of the specific candidate gene of choice. In order to test the efficiency of this system, we crossed the EN-line with a UAS-LacZ transgene or a background *Drosophila* strain for the transgenic lines (*w^1118^* or *yw*). As shown in Figure 3, the crosses containing Eaat1-Gal4, UAS-NICD, and UAS-LacZ (one copy of each transgene) showed a hypoxia tolerance similar to those containing only Eaat1-Gal4 and UAS-NICD (60%–80%), but significantly higher than those of controls (<25%) (*P* < 0.01), demonstrating that the presence of an additional UAS (Gal4 upstream activating sequence) did not significantly reduce the efficiency of Gal4-driven expression of UAS-NICD transgene, nor did it significantly affect the phenotype that is induced by NICD overexpression. Hence, the dosage of Eaat1-Gal4 is sufficient to simultaneously drive the expression of two UAS-transgenes.

### Evolutionarily conserved mechanisms underlying Notch-conferred hypoxia tolerance

It has been shown that combinatorial and context-dependent interactions between cellular signaling pathways regulate a wide and diverse range of biological processes for development, homeostasis, and disease. Previous studies have demonstrated that cellular context-specific integration of Notch signaling with other genes and pathways are essential for mediating the action of Notch in various developmental and pathological processes (Hurlbut *et al.* 2007; Bray and Bernard 2010; Borggrefe and Liefke 2012). However, the specific mechanisms by which Notch regulates hypoxia survival still largely remain uncharacterized.

**Figure 4:**
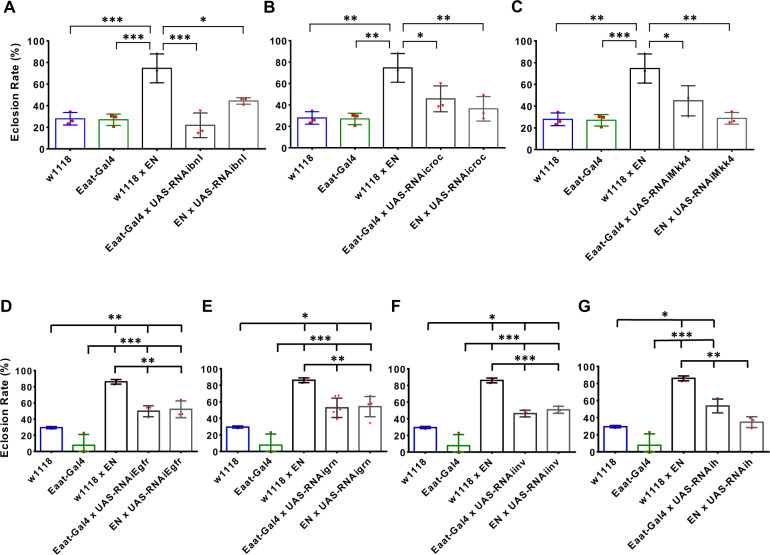
Genetic interactions regulating hypoxia tolerance conferred by activating Notch in Eaat1-positive glial cells. Specific UAS-RNAi line targeting bnl, croc, Egfr, grn, h, inv or Mkk4 was crossed with the EN line to knock down the expression of the targeted genes individually on the background of Notch activation in the Eaat1-positive glial cells. The eclosion rate of the progeny under hypoxic condition (5% O_2_) were measured. A significant reduction of Notch activation-conferred hypoxia survival was observed in the crosses with (A) bnl knock down [Ordinary one-way ANOVA; *F*_(4,10)_ = 18.25, *P* = 0.0001], (B) croc knock down [Ordinary one-way ANOVA; *F*_(4,10)_ = 11.09, *P* = 0.0011], (C) Mkk4 knock down [Ordinary one-way ANOVA; *F*_(4,10)_ = 13.44, *P* = 0.0005], or (G) h knock down [*F*_(4,10)_ = 44.24, *P* < 0.0001]. In contrast, knock down (D) Egfr [Ordinary one-way ANOVA; *F*_(4,10)_ = 37.23, *P* < 0.0001), (E) grn [Ordinary one-way ANOVA; *F*_(4,10)_ = 23.66, *P* < 0.0001]or (F) inv [Ordinary one-way ANOVA; *F*_(4,10)_ = 56.93, *P* < 0.0001]did not significantly diminish the hypoxia survival rate to control levels. Bars represent mean ± SD (*n *= 3 to 6 vials) for each group (*P < 0.05, ** *P* < 0.01, ***P < 0.001; Turkey’s multiple comparison tests).

To identify the genetic interactions regulating Notch-conferred hypoxia tolerance in Eaat1-positive glial cells, we tested 7 of the 23 evolutionarily conserved candidate genes [*i.e., branchless* (*bnl*), *crocodile* (*croc*), *Epidermal growth factor receptor* (*Egfr*), *grain* (*grn*), *hairy* (*h*), *invected* (*inv*), and *MAP kinase kinase 4* (*Mkk4*)] that have available VALIUM20 UAS-RNAi transgenic lines from the *Drosophila* Transgenic RNAi Project (TRiP) at Harvard Medical School. These TRiP RNAi lines contain a 21 bp targeting shRNAi sequence embedded into a micro-RNA (miR-1) backbone that is very effective for knocking down the expression of a target gene in both soma and germline (Ni *et al.* 2011; Perkins *et al.* 2015). As expected, knocking down *hairy*[*h*, one of the Notch downstream genes that regulates development and hypoxia tolerance in *Drosophila* (Kim *et al.* 2000; Cui 2005; Zhou *et al.* 2008)] decreased Notch activation-conferred hypoxia tolerance, reducing survival rate from 86.1% to 34.7% in hypoxia (*P* < 0.01) (Figure 4G). Furthermore, we found that knocking down *bnl*, *croc* and *Mkk4* also reduced Notch-conferred hypoxia tolerance (Figure 4, A–C). However, knocking down the other candidate genes (*Egfr*, *grn*, and *inv*) did not show a clear modifier effect, *i.e.*, knocking down *Egfr*, *grn*, and *inv* on the background of Notch activation ([*EN*] × [*UAS-RNAiEgfr*]; [*EN*] × [*UAS-RNAigrn*] and [*EN*] × [*UAS-RNAiinv*]) showed hypoxia survival rates that were still significantly higher than those of the controls (Figure 4, D–F).

The role of *bnl*, *croc* and *Mkk4* in *Drosophila* neuronal development has been reported in various studies. *Branchless* (*bnl*) is a fibroblast growth factor (*FGF*) homolog in *Drosophila* that is an important regulator of tracheal and neuronal morphogenesis (Barrett *et al.* 2008; Muha and Muller 2013;Du *et al.* 2017). A neuronal expression of *bnl* has been detected in embryo and larval brain, which is essential for the induction of cell migration and neuronal connection (Du *et al.* 2017). Our results indicate that *bnl* is also expressed in the Eaat1-positive glial cells in addition to neurons. This pattern of expression may be important in neuronal development through regulating cell migration and axon outgrowth to create functional neuronal networks. Furthermore, the expression of *bnl* is highly inducible by hypoxia, and such expression of *bnl* is important to trigger the growth of the tracheal system to improve O_2_ delivery (Jarecki *et al.* 1999; Centanin *et al.* 2008). Becauseinteractions between *FGF* and Notch signaling has been observed in *Drosophila* and mammals in particular in the neuronal system (Bartlett *et al.* 1998; James *et al.* 2004; Yoon *et al.* 2004; Ghabrial and Krasnow 2006; Zhou and Armstrong 2007; Fujita *et al.* 2011; Voelkel *et al.* 2014), the current results suggest that bnl produced by the Eaat1-positive glial cells is essential for Notch-activation-conferred survival under severe hypoxic conditions, at least in part, by maintaining axon growth and integrity of proper neuronal-glial connectivity during development (Figure 6).

The *crocodile* (*croc*) gene encodes a member of the forkhead transcription factor family in *Drosophila*. Genes encoding this family of transcription factors are widely conserved during evolution ranging from yeast to humans (Hannenhalli and Kaestner 2009). In *Drosophila*, the expression of *croc* is mainly detected in embryo, third instar larvae and adult female flies (Lee and Frasch 2004; Lopez *et al.* 2017). At the embryonic stage, it is expressed in the head anlagen of the blastoderm and controls the establishment of head structures (Hacker *et al.* 1995). Although signaling mechanisms regulating the activity of croc are yet uncharacterized, our results indicate that the *Notch*/*croc* interaction within the Eaat1-positive glial cells is essential for organismal survival under hypoxic condition, possibly through maintaining the viability and function of these Eaat1-positive glial cells and, thus, neuronal development (Figure 6).

**Figure 5:**
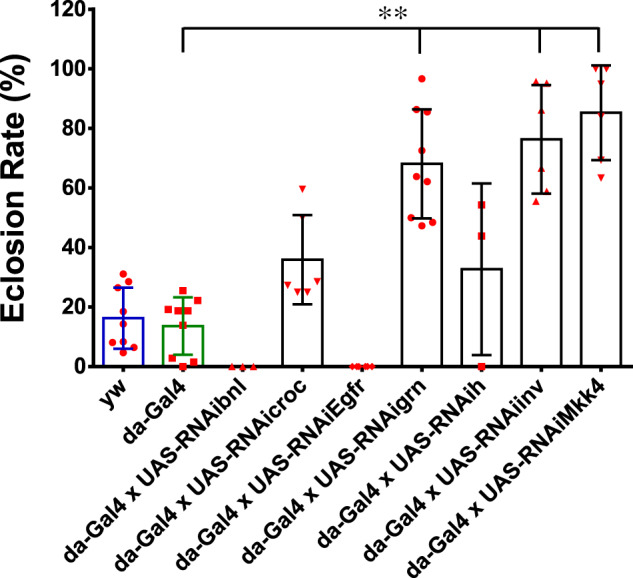
Effect of ubiquitous knocking down of candidate genes on hypoxia tolerance. Specific UAS-RNAi line targeting bnl, croc, Egfr, grn, h, inv, or Mkk4 was crossed with the da-Gal4 driver to ubiquitously knock down the expression of the targeted genes individually in *D.melanogaster*. The eclosion rate of the progeny under 5% O_2_ hypoxic condition was measured. A significant enhancement of hypoxia survival was detected in the flies with specific knocking down of grn, inv, and Mkk4. In contrast, the crosses with croc and h knocking down showed a similar survival rate with the controls. And ubiquitous knocking down of bnl or Egfr was lethal. Bars represent means ± SD (*n* = 3 to 6 vials) for each cross. Ordinary one-way ANOVA [*F*_(8,48)_ = 31.93, *P* < 0.0001]with Turkey’s multiple comparison tests (**P < 0.01).

**Figure 6:**
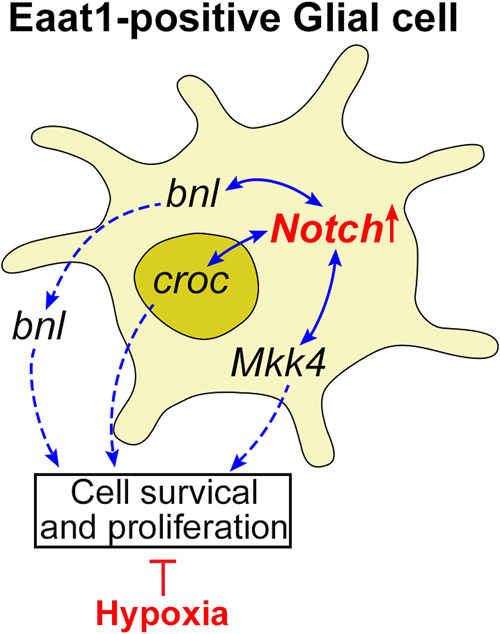
Schematic illustration of hypothetical functional output of Notch/bnl, Notch/croc and Notch/Mkk4 interactions under hypoxia. Hypoxia tolerance conferred by Notch activation in the Eaat1-positive glial cells requires functional synchronization with bnl, croc and Mkk4 to regulate cell survival and proliferation, such functional synchronization is essential for organismal survival under prolonged severe hypoxic conditions.

A number of studies have shown that Mkk4 regulates the activity of JNK pathway and neuronal development in *Drosophila*(Geuking *et al.* 2009; Rallis *et al.* 2010).The interactions between Notch and JNK signaling have been reported in *Drosophila* and mammals during development or under disease conditions. Such interactions may determine or fine-tune the final output of Notch signaling in a cell context-dependent fashion (Zecchini *et al.* 1999; Kim *et al.* 2005, 2010; Curry *et al.* 2006; Ho *et al.* 2015). It was very interesting to find that cell-specific and ubiquitous manipulation of *Mkk4* exhibited opposite phenotypes in terms of hypoxia tolerance (*i.e.*, knocking down Mkk4 specifically in Eaat1-positive glial cells led to lethality, but ubiquitous knocking down this gene enhanced hypoxia survival) (Figures 4C and 5), implying a cell context-dependent role of JNK signaling in hypoxia response. Indeed, it has been shown in tumor cells that hypoxia-induced JNK activation increases resistance to chemotherapeutic treatment (Comerford *et al.* 2004), but, in the pulmonary arteries, such activation leads to structural remodeling (Jin *et al.* 2000). Furthermore, we found that ubiquitous knocking down of several genes modulating the activity of JNK signaling can enhance hypoxia survival (Azad *et al.* 2012), which suggests a cell context-dependent role of JNK signaling on hypoxia response in the organism. Therefore, we hypothesize that the interaction between Mkk4 and Notch may regulate survival of glial cells under hypoxic conditions, and, hence, hypoxia tolerance in *Drosophila* (Figure 6).

In addition, we performed an experiment with ubiquitous knocking down of these candidate genes using the da-Gal4 driver to evaluate their role in *Drosophila* development and hypoxia tolerance (Figure 5). We found that the progeny of [*da-Gal4*] × [*UAS-RNAibnl*] or [*da-Gal4*] × [*UAS-RNAiEgfr*] crosses were lethal under both normoxia and hypoxia conditions. In contrast, flies with da-Gal4>UAS-RNAigrn, da-Gal4>UAS-RNAiinv or da-Gal4>UAS-RNAiMkk4 exhibited significantly enhanced tolerance to hypoxia with > 2-fold eclosion rates as compared to those of the controls under hypoxia (*p* < 0.01). The result of *Mkk4* KD is somewhat surprising: unlike specific knocking down in the Eaat1-positive glial cells (Eaat1-Gal4>UAS-RNAiMkk4) (Figure 4C), ubiquitous knockdown of *Mkk4* significantly enhanced hypoxia tolerance in *Drosophila*, suggesting a developmental and/or cell type specific role of *Mkk4* in regulating organismal response to hypoxia.

## Conclusions and perspectives

In conclusion, we evaluated the interactions between Notch signaling and a group of evolutionarily conserved genes in Eaat1-positive glial cells and the role of such interactions in regulating hypoxia survival in *Drosophila*. The synergy of signaling between Notch and bnl, Notch and croc and between Notch and Mkk4 were found to be essential for Notch activation-conferred hypoxia tolerance. We hypothesize that the synergy between these functionally distinct signaling mechanisms regulates cell survival and neuronal development under hypoxic conditions in the *Drosophila* central nervous system, and this is also sufficient for the survival of the organism as a whole (Figure 6). Furthermore, we provided evidence indicating that *bnl*, *croc*, and *Mkk4* are expressed in the Eaat1-positive glial cells. These conserved mechanisms have high potential to be translated into humans for developing strategies to treat hypoxia-induced injuries in the central nervous system.

## Data availability

*D.melanogaster* strains are available upon request. The authors affirm that all data necessary for confirming the conclusions of the article are present within the article, figures, and tables.

Supplementary material is available at G3 online.

## Supplementary Material

jkab038_Supplementary_Data
