# Supplementary material for: Genetic interactions regulate hypoxia tolerance conferred by activating Notch in excitatory amino acid transporter 1-positive glial cells in Drosophila melanogaster
Source: G3 (Bethesda). 2021 Jan 28;11(2):jkab038. doi: 10.1093/g3journal/jkab038 (PMC8022968; doi:10.1093/g3journal/jkab038)
Supplement: jkab038_Supplementary_Data [file jkab038_supplementary_data.pptx]

## Slide 1
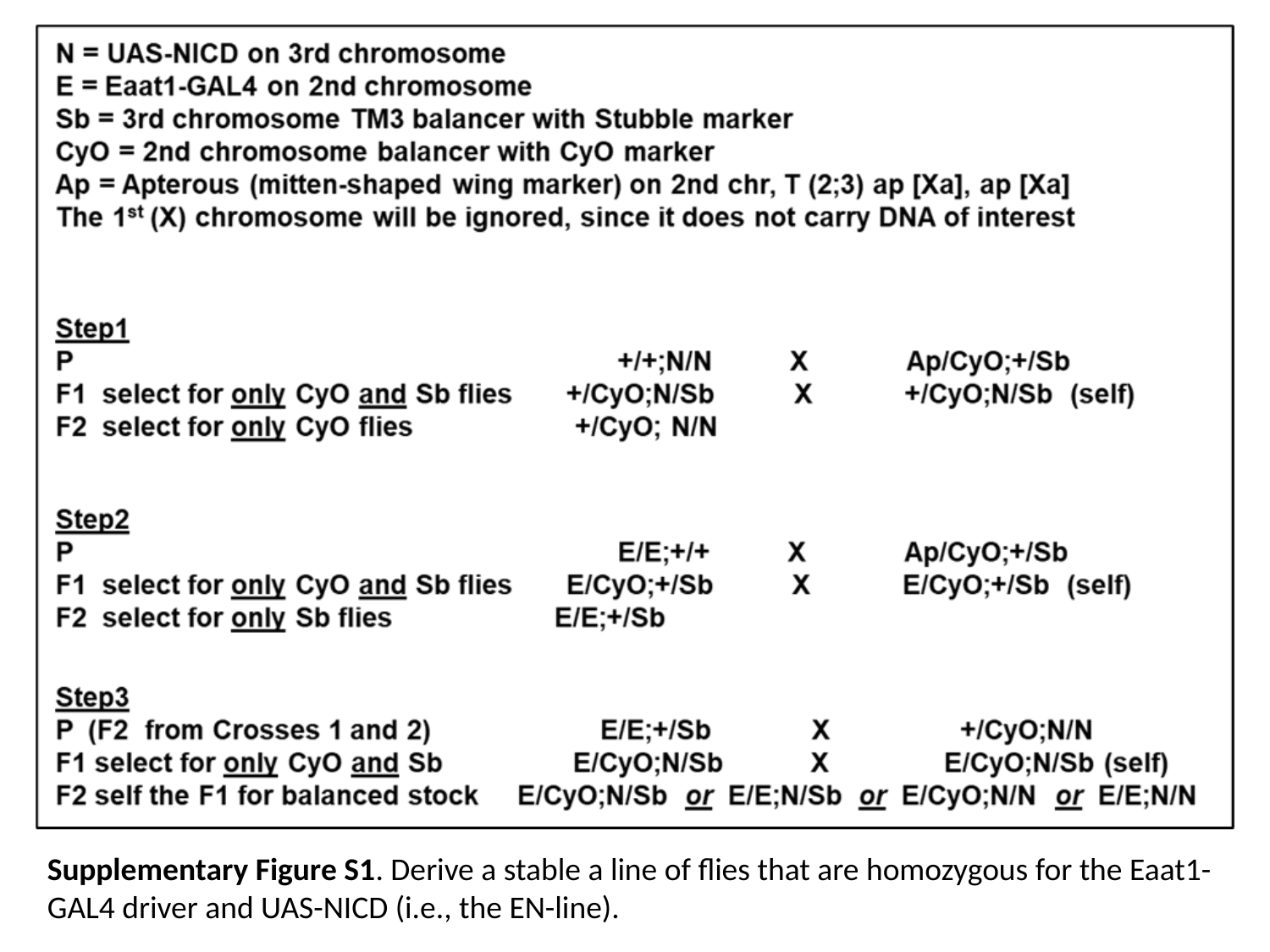

Supplementary Figure S1. Derive a stable a line of flies that are homozygous for the Eaat1-GAL4 driver and UAS-NICD (i.e., the EN-line).
